# Evaluation of Critical Quality Attributes of a Pentavalent (A, C, Y, W, X) Meningococcal Conjugate Vaccine for Global Use

**DOI:** 10.3390/pathogens10080928

**Published:** 2021-07-23

**Authors:** Barbara Bolgiano, Eilís Moran, Nicola J. Beresford, Fang Gao, Rory Care, Trusha Desai, Ida Karin Nordgren, Timothy R. Rudd, Ian M. Feavers, Prashant Bore, Sushil Patni, Vinay Gavade, Asha Mallya, Sameer Kale, Pankaj Sharma, Sunil K. Goel, Sunil Gairola, Suhas Hattarki, Nikhil Avalaskar, Annamraju D. Sarma, Marc LaForce, Neil Ravenscroft, Lakshmi Khandke, Mark R. Alderson, Rajeev M. Dhere, Sambhaji S. Pisal

**Affiliations:** 1 National Institute for Biological Standards and Control, South Mimms, Potters Bar EN6 3QG, UK; Eilis.Moran@mhra.gov.uk (E.M.); Nicola.Beresford@nibsc.org (N.J.B.); Fang.Gao@nibsc.org (F.G.); Rory.Care@nibsc.org (R.C.); Trusha.Desai@nibsc.org (T.D.); Karin.Nordgren@nibsc.org (I.K.N.); Tim.Rudd@nibsc.org (T.R.R.); ian.feavers@icloud.com (I.M.F.); 2 Serum Institute of India Pvt. Ltd., Hadapsar, Pune 411028, India; prashant.bore@seruminstitute.com (P.B.); sushil.patni@seruminstitute.com (S.P.); vinay.gavade@seruminstitute.com (V.G.); asha.mallya@seruminstitute.com (A.M.); sameer.kale@seruminstitute.com (S.K.); pankaj.sharma@seruminstitute.com (P.S.); sunil.goel@seruminstitute.com (S.K.G.); sunil.gairola@seruminstitute.com (S.G.); suhas.hattarki@seruminstitute.com (S.H.); a.nikhil@seruminstitute.com (N.A.); ad.sarma@seruminstitute.com (A.D.S.); fmarclaforce@gmail.com (M.L.); rajeev.dhere@seruminstitute.com (R.M.D.); sspisal@seruminstitute.com (S.S.P.); 3 Department of Chemistry, University of Cape Town, Rondebosch, Cape Town 7701, South Africa; Neil.Ravenscroft@uct.ac.za; 4 Center for Vaccine Innovation and Access, PATH, Seattle, WA 98121, USA; lkhandke@path.org (L.K.); malderson@path.org (M.R.A.)

**Keywords:** *Neisseria meningitidis*, glycoconjugates, meningitis, adjuvant, chromatography, immunogenicity, polysaccharide, serum bactericidal, acetylation, nuclear magnetic resonance spectroscopy

## Abstract

Towards achieving the goal of eliminating epidemic outbreaks of meningococcal disease in the African meningitis belt, a pentavalent glycoconjugate vaccine (NmCV-5) has been developed to protect against *Neisseria meningitidis* serogroups A, C, Y, W and X. MenA and X polysaccharides are conjugated to tetanus toxoid (TT) while MenC, Y and W polysaccharides are conjugated to recombinant cross reactive material 197 (rCRM_197_), a non-toxic genetic variant of diphtheria toxin. This study describes quality control testing performed by the manufacturer, Serum Institute of India Private Limited (SIIPL), and the independent control laboratory of the U.K. (NIBSC) on seven clinical lots of the vaccine to ensure its potency, purity, safety and consistency of its manufacturing. In addition to monitoring upstream-manufactured components, samples of drug substance, final drug product and stability samples were evaluated. This paper focuses on the comparison of the vaccine’s critical quality attributes and reviews key indicators of its stability and immunogenicity. Comparable results were obtained by the two laboratories demonstrating sufficient levels of polysaccharide *O*-acetylation, consistency in size of the bulk conjugate molecules, integrity of the conjugated saccharides in the drug substance and drug product, and acceptable endotoxin content in the final drug product. The freeze-dried vaccine in 5-dose vials was stable based on molecular sizing and free saccharide assays. Lot-to-lot manufacturing consistency was also demonstrated in preclinical studies for polysaccharide-specific IgG and complement-dependent serum bactericidal activity for each serogroup. This study demonstrates the high quality and stability of NmCV-5, which is now undergoing Phase 3 clinical trials in Africa and India.

## 1. Introduction

Due to the global burden of vaccine-preventable meningitis and other related diseases caused by pathogenic, encapsulated bacteria primarily colonizing the respiratory tract, there is a continuing need to develop and provide vaccines suitable for regional needs [1]. The Global Burden of Disease Study 2016 recommended increasing the breadth of coverage of vaccines [2]. This is particularly important, for the African meningitis belt, comprising 26 countries in sub-Saharan Africa from Senegal and The Gambia in the west to Ethiopia in the east, experience a disproportionately high share of meningococcal disease. Moreover, many low- and middle-income countries suffer threatening economic stability alongside the threat to human life and life prospects as a result of meningitis [3,4].

For the past two decades, high quality meningococcal glycoconjugate vaccines have been responsible for protection against significant mortality and morbidity from meningitis and septicemia [5]. Immunity generated through the development of T-cell dependent bactericidal antibody in those most at risk has resulted in reducing nasopharyngeal carriage and inducing herd immunity in highly vaccinated populations [6]. Vaccination of infants, toddlers and young adults ensures levels of circulating antibodies are adequate to recruit complement and target the Gram-negative bacterium, *Neisseria meningitidis*. The introduction of a meningococcal A (MenA) conjugate vaccine in the meningitis belt in 2010, initially in mass campaigns and subsequently in routine infant immunization programs [7], has led to the elimination of disease caused by serogroup A [8,9]. Epidemics due to serogroups C, W, and X meningococci have, however, continued in the meningitis belt countries after the introduction of MenA vaccine and are responsible for up to 60% of confirmed cases of disease [10,11,12,13]. In the first half of 2015, more than 9367 suspected cases and 549 deaths were associated with serogroup C infections in Niger alone [12]. In 2019, there were 15,324 suspected cases of meningitis during the meningitis season, with serogroup C the most dominant, comprising 33% of positive samples, followed by serogroup X (12%) and serogroup W (10%) [13]. In 2011, WHO’s Strategic Expert Advisory Group on Immunization expressed its concern about the lack of a vaccine against group X meningococci given the annual outbreaks caused by meningococci of this serogroup [14].

During the development of the monovalent MenA conjugate vaccine between 2002 and 2008, quadrivalent meningococcal conjugate vaccines were developed and initially licensed in high income countries. The first vaccine designed to protect against serogroups A, C, W and Y conjugated to a single carrier protein, diphtheria toxoid, was licensed in the US in 2005 [15]. Two other quadrivalent vaccines followed: CRM_197_ and TT conjugates, licensed in 2010 and 2012, and WHO prequalified for UNICEF procurement and use in global immunization programs in 2013 and 2016, respectively [16,17].

Following on from the Meningitis Vaccine Project’s development of MenAfriVac, the partnership between PATH and the Serum Institute of India Pvt Ltd. (SIIPL) is now developing an affordable pentavalent meningococcal conjugate vaccine (NmCV-5) for protection against disease caused by *N. meningitidis* serogroups A, C, Y, W and X, with financial support provided by the UK Foreign, Commonwealth & Development Office (formerly Department for International Development) [18]. For the pentavalent vaccine formulation, serogroup A and X polysaccharides are conjugated to TT, and serogroup C, W, and Y polysaccharides are conjugated to rCRM_197_, expressed in *Pseudomonas fluorescens* [19]. The vaccine is presented as a lyophilized product that is reconstituted with saline prior to immunization. A Phase 1 trial of NmCV-5 in healthy US adults (18–45 years of age) demonstrated that the vaccine is safe and well tolerated and elicits functional immune responses (complement-dependent serum bactericidal activity) against all 5 targeted serogroups [20]. This was the first clinical trial of a vaccine containing a MenX conjugate [21]. A Phase 2 study in toddlers (aged 12–16 months) in Mali was designed to confirm safety and immunogenicity and to select a formulation (adjuvanted or non-adjuvanted) for Phase 3 trials. The study results confirmed that NmCV-5 is safe and immunogenic against all serogroups and use of an aluminum phosphate adjuvant did not provide additional benefit [22]; hence, Phase 3 formulations of NmCV-5 are reconstituted with saline (without adjuvant). Phase 3 trials in Africa and India are ongoing.

There is a global requirement for meningococcal conjugate vaccines to meet ICH Guidelines Q5C, which outlines the stability requirements for the drug substances and drug product to establish shelf life [23]. Three consistency Phase 3 clinical trial NmCV-5 batches were fully evaluated by SIIPL, with independent confirmatory testing of key quality attributes being performed by the National Institute for Biological Standards and Control (NIBSC), U.K. Official lot release was performed by the Central Drug Laboratory, India’s National Control Laboratory. Prior to Phase 3, the process and analytical methods were validated to ensure consistent and well controlled product manufacturing. The manufacturing consistency was shown by ensuring the vaccine meets specifications and, in particular, key critical quality attributes for a conjugate vaccine such as the potency of the conjugate vaccines—that is, the ability to induce preclinical protective immunity through the production of bactericidal antibodies, which relies on the effective conjugation of the oligo- or polysaccharide to the carrier protein and the integrity of the vaccine molecule. Assays for stability-indicating markers, such as free saccharide and molecular size, are therefore important tools for assessing relationships between vaccine quality and immunogenicity.

## 2. Results

### 2.1. Critical Quality Attributes of Clinical Lots of NmCV-5 and Vaccine Components

**Figure 1:**
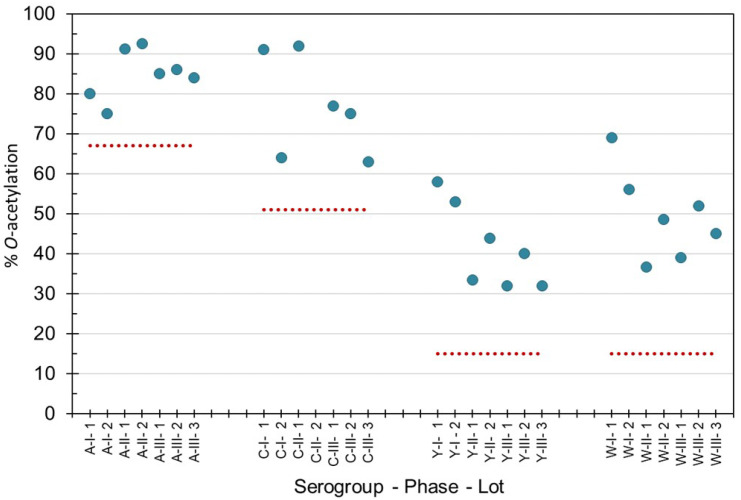
Percentage *O*-acetylation levels of purified capsular polysaccharide from meningococcal groups A, C, Y and W manufactured for Phase 1, 2 and 3 clinical and consistency lots of NmCV-5. ^1^H-NMR spectroscopy was performed at NIBSC to determine the mol *O*-acetyl/mol repeating unit. The dotted lines indicate the lower limits according to WHO Guidelines for meningococcal polysaccharides [24] and conjugates [25,26].

*O*-acetylation levels of purified bulk capsular polysaccharides manufactured for Phase 1, 2 and 3 campaigns were within the acceptable levels recommended by the World Health Organization (WHO) (Figure 1) for groups A, C, Y and W [24,25,26]. MenX polysaccharide is not *O*-acetylated. Phase 3 consistency lots contained an average (±std dev), 85 ± 1% *O*-acetylation (MenA), 72 ± 8% (MenC), 35 ± 5% (MenY) and 45 ± 7% (MenW), as determined by ^1^H-NMR at NIBSC. Using the Hestrin method at SIIPL, the consistency lots contained on average *O*-acetyl of 92 ± 5% *O*-acetylation (MenA), 77 ± 4% *O*-acetylation (MenC), 33 ± 7% *O*-acetylation (MenY) and 45 ± 8% *O*-acetylation (MenW). Combining the Phase 3 lot data from both laboratories gave a standard deviation of ≤6% *O*-acetylation demonstrating the consistency of the lots and close correlation between the laboratories’ data. The polysaccharides complied with pharmacopeial requirements for all impurities and endotoxin content [24,25,26].

**Figure 2:**
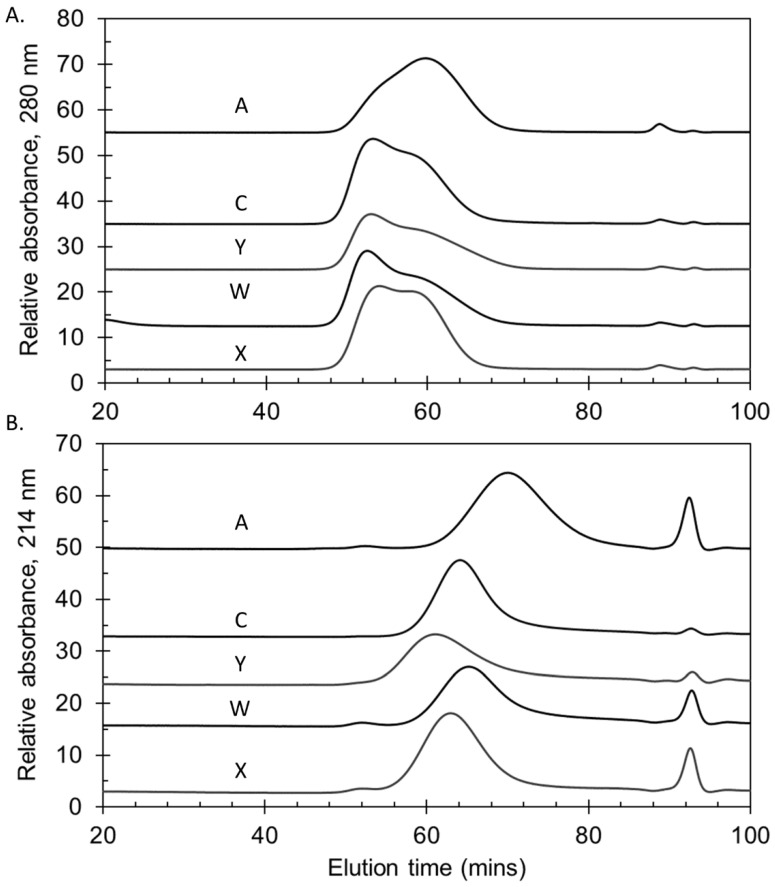
Molecular size chromatograms of representative lots of group A, C, Y, W and X polysaccharides (**A**) and bulk conjugates (**B**) manufactured for Phase 3 trials with NmCV-5. A TSK 6000 + 5000_PWXL_ column series was used with a PBS (pH 7.4) buffer. Vo and Vt markers eluted at 46.2 min and 99.2 min, respectively. Data were obtained at NIBSC.

The polysaccharides manufactured for the Phase 3 trials were consistent in molecular size, with representative lots shown in Figure 2A. In common with other sources of meningococcal polysaccharides, serogroup A polysaccharide was relatively smaller than the other serogroups [27]. NIBSC and SIIPL both measured the percent of material eluting by K_D_ 0.50, the mid-point of the column series, as a quantitative way of measuring size. Combining the Phase 3 lot data from the two laboratories gave mean (±standard deviation) values of 79 ± 5% (MenA), 91 ± 2% (MenC), 93 ± 3% (MenY), 88 ± 2% (MenW) and 96 ± 1% (MenX), demonstrating the consistency of the lots and close correlation between the laboratories’ data (overall ≤6% CV).

The molecular sizing chromatograms for the MenA and MenX bulk conjugates were typical of TT conjugates (A and X), with a broad size distribution with multiple peaks. The bulk conjugates made with rCRM_197_ conjugated to polysaccharide were of a similar size as the TT conjugates (Figure 2B). This was more evident using the more accurate SEC-MALS method that measures M_w_ (weight-average molar mass, g/mol) based on light scattering, rather than the column matrix-based elution of M_w_ standards. M_w_ values of 4.52 × 10^6^ to 4.66 × 10^6^ g/mol (MenA); and, 5.70 × 10^6^ to 7.47 × 10^6^ g/mol (MenX) for the TT conjugates; and, 7.06 × 10^6^ to 1.14 × 10^7^ g/mol (MenC); 6.25 × 10^6^ to 1.10 × 10^7^ g/mol (MenY); and, 7.05 × 10^6^ to 8.02 × 10^6^ g/mol (MenW) for the rCRM_197_ conjugates. The rCRM_197_ conjugates made with polysaccharides are larger and more polydisperse than those made with oligosaccharides [28,29,30]. The percent eluting by K_D_ 0.50 determined at SIIPL for the three Phase 3 lots were: 96 ± 1% (MenA), 94 ± 1% (MenC), 93 ± 1% (MenY), 94 ± 1% (MenW) and 96 ± 1% (MenX).

**Figure 3:**
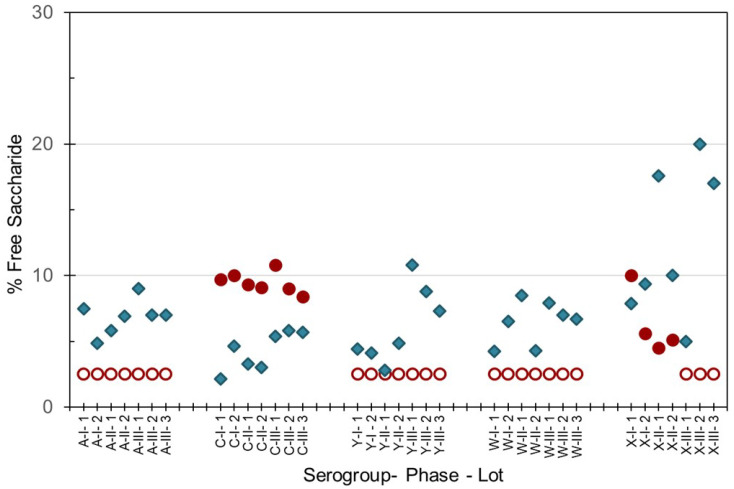
Free saccharide content of bulk conjugates from meningococcal groups A, C, Y, W and X manufactured for Phase 1, 2 and 3 clinical and consistency lots of NmCV-5. Free saccharide was separated from conjugated saccharide using DOC-precipitation at SIIPL (♦) or ultrafiltration at NIBSC (●, **◯**). Open circles denote lower limits of quantification. Saccharide concentrations were determined by HPAEC-PAD using polysaccharide standards.

As the integrity of the conjugated saccharides must be maintained through the manufacturing process, percent free saccharide and saccharide contents were hence measured in both the monovalent bulk conjugates and multivalent final drug product. The method of separation of the non-conjugated or ‘free’ saccharide from protein-conjugated saccharides gave slightly different results. Slightly higher values were obtained with the DOC-HCl protein-precipitation method (SIIPL) compared to the ultrafiltration size-dependent method (NIBSC), with the exception of MenC and MenX as shown in Figure 3. Up to 15% free saccharide values were determined in MenA, C, Y and W monovalent bulk conjugates, and up to 20% free saccharide was measured in MenX bulk conjugates. Free saccharide in the pentavalent NmCV-5 was less than 30% (data not shown).

The endotoxin content of Phase 3 vaccine lots was on average (±std dev) 207 ± 25 IU/dose by the kinetic turbidometric assay (KTA) method (SIIPL), or 200 ± 87 IU/dose by the semi-quantitative Gel-clot method (NIBSC), both which use Limulus amebocyte lysate (LAL). The conjugate bound to aluminum phosphate adjuvant was analyzed and the data showed that each of the serogroup polysaccharide conjugates were partially bound: MenA (20% bound), MenC (61%), MenY (24%), MenW (24%) and MenX (19%).

### 2.2. Stability of Monovalent Drug Substances and NmCV-5 Drug Product

**Figure 4:**
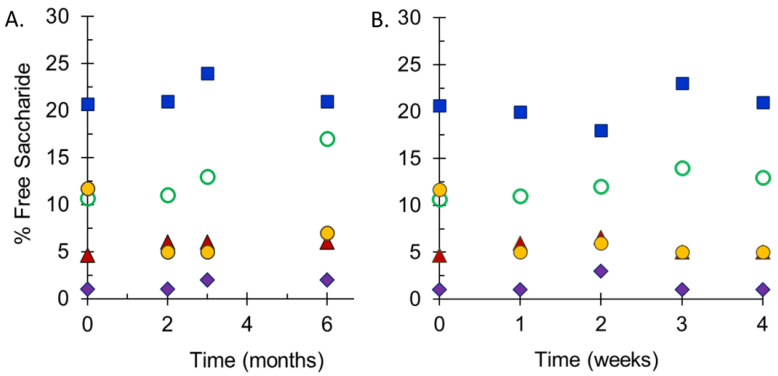
Free saccharide content of stability samples of NmCV-5 Phase 3 clinical and consistency lots. Samples were stored at 25 °C for up to 6 months (**A**) or at 40 °C for up to 4 weeks (**B**). Free non-conjugated saccharide was separated from conjugated saccharide using DOC-precipitation. Saccharide concentrations of groups A (♦), C (▲), Y (**◯**), W (●) and X (■) were determined by HPAEC-PAD using polysaccharide standards. Data were obtained by SIIPL and are from representative clinical consistency lots.

Stability studies performed at SIIPL monitored the integrity of conjugate vaccines maintained at 2–8 °C and at elevated temperatures by measurement of free saccharide for both the drug substances and drug product and molecular size distribution for the drug substances. Following accelerated stability studies at 25 °C for 6 months (Figure 4A), or 40 °C for 4 weeks (Figure 4B), the free saccharide content in freeze-dried pentavalent vials was relatively stable. MenA had the lowest percent free saccharide values in each study (<5%), while MenX had the highest (15–25%), reflecting the values in the starting materials. There were slight increases in free saccharide at the elevated temperatures, but these were within the expected variability of the assay. The maximum increase in free saccharide for each serogroup was similar at both elevated temperatures. In addition to these studies, elevated temperature sample analysis at NIBSC demonstrated that the lyophilized final vial form of the vaccine was stable and there was no evidence of instability of any of the meningococcal serogroup polysaccharide constituents at 25 °C for up to 6 months, or 40 °C for 24 weeks. Additionally ECTC studies performed by SIIPL i.e., 40 °C for 12 weeks after storage at 2–8 °C for 36 months also confirmed the stability of NmCV-5 (data not shown).

**Figure 5:**
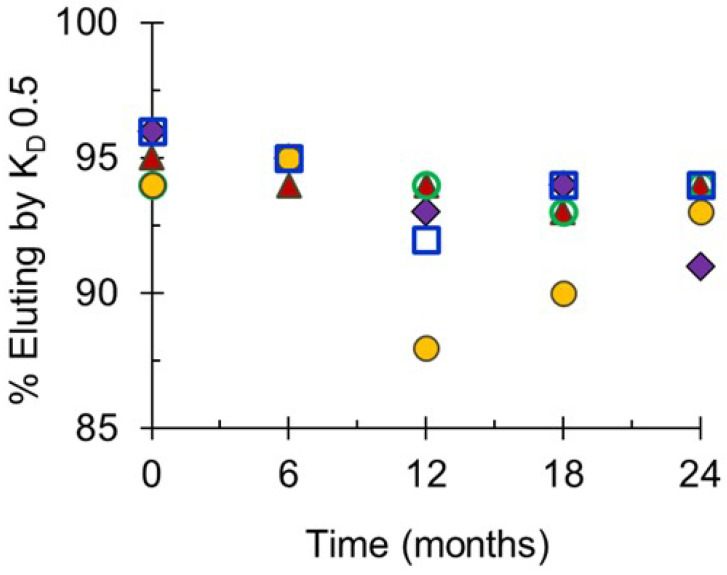
Molecular size distribution of monovalent bulk conjugates for Phase 3 lots of NmCV-5 stored at −20 °C for up to 24 months. Molecular size distributions of serogroups A (♦), C (▲), Y (**◯**), W (●) and X (**☐**) were determined using TSK 6000 + 5000_PWXL_ column series in PBS (pH 7.4). Data were obtained at SIIPL and are from representative clinical consistency lots.

Molecular sizing profiles based on size exclusion HPLC analyses demonstrated that the monovalent bulk conjugates were stable at the designated storage temperature of −20 °C for up to 24 months before formulation, filling and freeze-drying (Figure 5). The slight decreases in the percent eluting by the K_D_ of 0.50 over the course of the study were minimal and within the expected assay variation. At NIBSC, molecular sizing profiles of combined final lots stored at the designated storage temperature of 2–8 °C for up to 32 months (for Phase 2 lots) or 14–15 months (for Phase 3 lots) showed no evidence of degradation by UV or RI detection, and there were no decreases in the specified elution values corroborating the evidence that the conjugates in the freeze-dried final vial were stable (data not shown).

## 3. Preclinical Immune Responses to NmCV-5

### 3.1. Serogroup-Specific Polysaccharide Responses in Mice to a Pentavalent R&D Vaccine Formulation

The aim of this preclinical study was to: (1) demonstrate the immunogenicity of conjugated MenX polysaccharide; (2) assess the effect of aluminum phosphate adjuvant on each antigen; and (3) compare the immunogenicity in mice for each of the other serogroups with that of a licensed meningococcal conjugate vaccine. A strong immune response to MenX conjugated to TT was shown by a 100-fold rise in serum IgG levels following a second dose compared to baseline titers, and a further increase in antibody concentration was observed after a third dose; statistical significance was only achieved in the adjuvanted group (*p* < 0.05). The use of 1/10 SHD (single human dose) allowed for discrimination between immunization number (dose number) and vaccine formulation.

**Figure 6:**
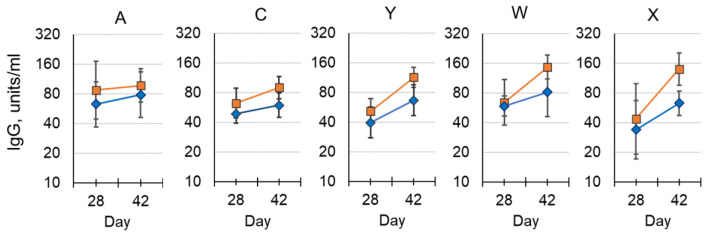
Immunogenicity of NmCV-5 in BALB/c mice (EIGHT per group). Post dose-2 and post dose-3 polysaccharide-specific IgG from a pentavalent R&D lot were administered either with (■) or without (♦) aluminum phosphate adjuvant. The geometric mean of serotype-specific IgG concentrations in units/mL, were determined by ELISA. Error bars represent 95% confidence intervals. The study was performed at NIBSC.

The addition of aluminum phosphate adjuvant led to an increase in antibody concentration at all time points for all serogroups, although only MenC, MenW and MenX following the third dose achieved statistical significance (*p* < 0.05; Figure 6). No significant difference in antibody concentrations was observed for other serogroups between the pentavalent vaccine and the control group that received a MenACWY-CRM_197_ conjugate vaccine, after receiving three doses. However, following the second dose there was a significant increase for MenY and MenW in both pentavalent vaccine preparations above the control group (data not shown).

### 3.2. Serogroup-Specific IgG and Bactericidal Antibody Responses in Rabbits to NmCV-5 Clinical Lots

The immunogenicity studies in animals were performed to evaluate the vaccine lots manufactured for use in clinical trials, including: (1) a Phase 1 NmCV-5 lot with and without adjuvant; and (2) Phase 3 NmCV-5 consistency lots without adjuvant, in New Zealand White rabbits.

**Figure 7:**
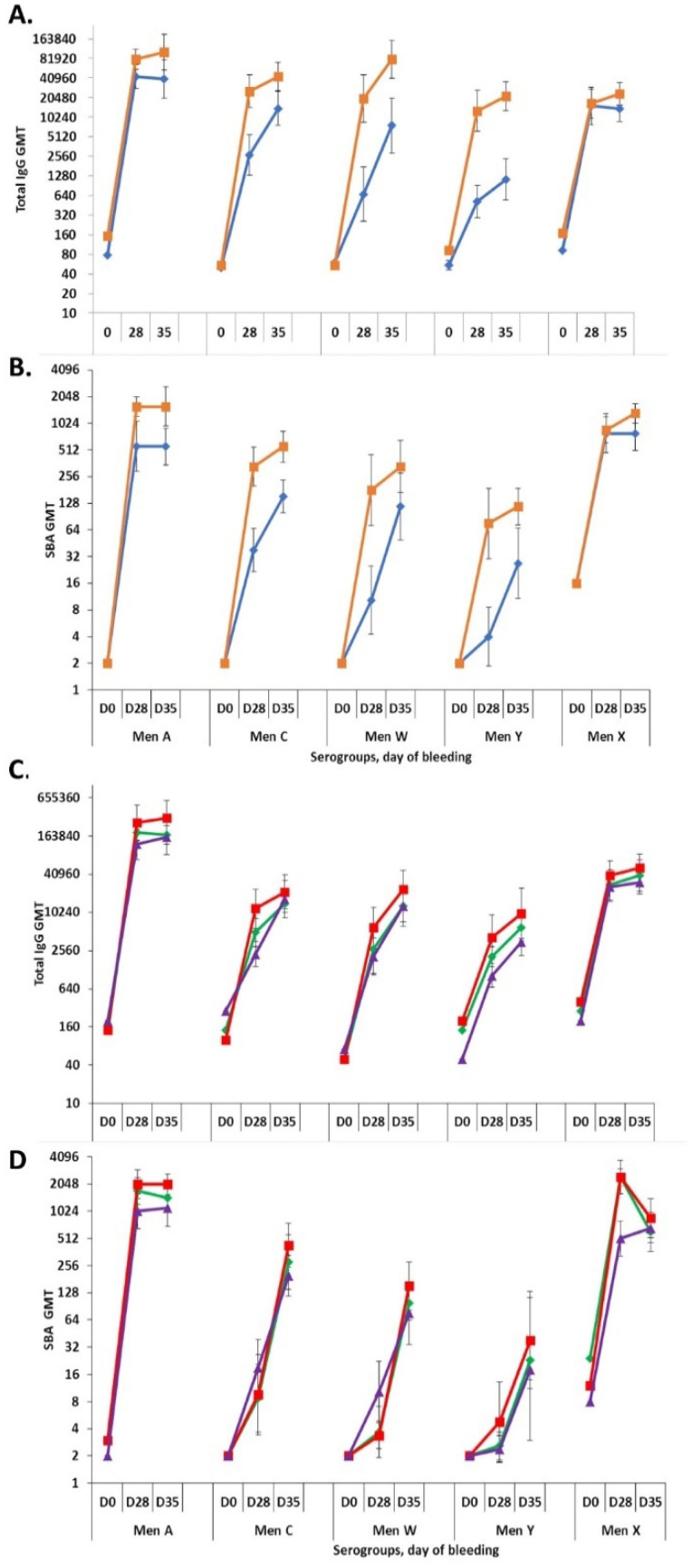
Immunogenicity study in New Zealand White rabbits (eight/formulation) of NmCV-5 Phase 1 (**A**,**B**), and Phase 3 (**C**,**D**) clinical lots. Total IgG (**A**,**C**) and rabbit complement-dependent serum bactericidal activity titers (**B**,**D**) to meningococcal serogroups A, C, Y, W, and X in serum samples collected at three timepoints (Days 0, 28, and 35) were determined using the bead-based ELISA and rSBA assay. The immunogenicity of Phase 1 vaccine lots were tested as adjuvanted (■) and non-adjuvanted (♦) formulations (**A**,**B**). Phase 3 clinical consistency lots (n = 3). Lot 1 (♦), Lot2 (■), Lot3 (▲) were non-adjuvanted based upon data from the Phase 1 and 2 clinical trials [20,22] (**C**,**D**). Results are expressed as Geometric Mean Titers. Error bars represent 95% confidence intervals. Statistical significance between the adjvuanted and non-adjuvanted groups is described in the text. The study was performed at SIIPL.

For the Phase 1 formulation lot, IgG titers for serogroups A and X were found to be comparable in formulations with and without aluminum phosphate adjuvant, whereas the titers for C, Y and W were found to be higher in the formulation with adjuvant (*p* < 0.05; Figure 7A). The functional SBA titers for serogroups W and X were comparable with and without adjuvant, whereas the titers for A, C and Y were found to be higher in the formulation with aluminum phosphate (*p* < 0.05; Figure 7B). For the three Phase 3 lots, all without adjuvant, all formulations elicited comparable IgG and functional SBA titers for all five serogroups and time points (Figure 7C,D).

During development of the vaccine, a study was performed in rabbits to assess the effect of the novel MenX component on the immunological response to the other serogroups. The inclusion of serogroup X capsular polysaccharide conjugate in the pentavalent formulation demonstrated less than 2-fold differences in titers to the other serogroups in comparison to A, C, Y and W titres obtained with the quadrivalent formulation.

## 4. Discussion

The optimal control of meningococcal disease in sub-Saharan Africa requires the implementation of a multivalent conjugate vaccine that provides protection against all the main disease-causing serogroups, including serogroup X. The poorly immunogenic MenX polysaccharide [31], when conjugated to a TT carrier protein, elicited strong immune responses in mice and rabbits, either alone or in combination with 4 other conjugates, comprised of polysaccharide conjugated either to TT (MenA) or to rCRM_197_ (MenC, Y and W) [20,22]. The strong immunogenicity of MenX-TT was also replicated in a Phase 1 trial in US adults [20] and a Phase 2 trial in Malian toddlers [22].

Extensive research to identify optimal production strains, most efficient conjugation chemistries, carrier protein combinations that produce the most immunogenic conjugates, and optimal formulation led to the development of the freeze-dried pentavalent vaccine that has completed Phase 1 and 2 clinical studies [20,22] and is now being evaluated in Phase 3 trials in Africa and India. Assessment of purified polysaccharides and conjugates by ^1^H-NMR was initially conducted at the University of Cape Town to confirm structural composition, conjugation markers and *O*-acetylation, and identify any residual impurities [32]. Analytical method development for vaccine combinations containing polysaccharides with some structural similarities [33], is a challenge, and encompassed comparisons of ELISA (and multiplexed ELISA) approaches with established methods for verifying identity and specificity, total saccharide content and free saccharide levels. Collaboration between the manufacturer, SIIPL, and an independent control laboratory was invaluable for a bi-directional sharing of methods, reagents, standards, and results from complementary approaches; materials from R&D lots through to Phase 3 lots were evaluated at NIBSC.

Different methods for *O*-acetylation determination, free saccharide determination, and endotoxin content measurement provided added value. Where similar methods were chosen, a close correlation of results between laboratories gave evidence of inter-lot consistency, important for comparisons between manufacturing campaigns and scale-ups, and critically, the Phase 3 consistency lots. While ^1^H-NMR spectroscopy gives the most accurate and precise values for *O*-acetylation quantitation, Hestrin-determined values were precise and in close agreement with ^1^H-NMR measurements. The two methods for percent free saccharide determination gave slightly different values with some serogroup bias. There may be advantages to using the DOC-HCl precipitation method which depends on precipitation of the conjugated protein, as the ultrafiltration method relies on the efficiency of size-based separation and may be giving an underestimate for some serogroups (A, Y and W). For serogroups C and X, the ultrafiltration method gave similar or higher values than the protein precipitation method. For measuring endotoxin content, the KTA method is quantitative and gave more precise values than the semi-quantitative gel clot method. Another lesson learned was that continual comparison and a common approach to saccharide content determination, in this case identical HPAEC-PAD methods, and use of polysaccharide standards was very important. During the course of the project, new WHO International Standards for MenA, X, W and Y polysaccharides were established. Comparison of monoclonal antibody reactivities used for identity assays was also critical.

The freeze-dried formulation in 5-dose vials that was evaluated in clinical trials was demonstrated to have remarkably high stability in terms of all five active ingredients, with integrity measured both at the level of the saccharide and the bulk conjugate by HPLC-SEC. In addition to the approaches outlined in this paper, many additional studies were performed at each scale of manufacturing. The stability of the vaccine for up to 8 h following reconstitution (i.e., in the clinical setting), and the stability under extended controlled temperature conditions at 40 °C at the end of shelf-life (i.e., outside the cold chain at the vaccination center) are currently being verified. These studies were performed for MenAfriVac prior to an application to WHO for a controlled temperature chain (CTC) indication for its use while being delivered and used at final destinations without the need for cold storage [34,35,36]. Such studies are ongoing with NmCV-5.

The demonstration of functional antibody responses following two doses of NmCV-5 in rabbits, increasing after a third dose, provided confidence that adequate immunogenicity compared to a licensed quadrivalent NmCV could be achieved in clinical trials. Importantly, the test strains used for the SBA assays were identical to those used for evaluation of clinical sera, and the *O*-acetylation-expressing status of the strains were known for serogroups A, C and Y. Patterns of polysaccharide-specific IgG responses typically associated with conjugate vaccines were obtained for each serogroup both in mouse and rabbit models of immunogenicity.

Both species of animals tested responded to most serogroups better when aluminum phosphate adjuvant was present, but this only reached statistical significance for some. For rabbits, adjuvant impacted positively on the production of IgG and bactericidal antibodies for serogroups C and Y. In mice, the MenX response was significantly higher with adjuvant, but there was not a consistently higher increase with the other serogroups. These indicators of a potentially positive impact of adjuvant were sufficient to justify the inclusion of an adjuvant arm in the Phase 1 and 2 clinical trials. Neither human adults nor toddlers, however, were shown to benefit from the inclusion of the adjuvant [20,22], and thus Phase 3 trials are being conducted with the non-adjuvanted vaccine. Notably, none of the three WHO-prequalified quadrivalent vaccines include an adjuvant [15,16,17].

The conjugation of polysaccharide to rCRM_197_ using cyanylation and linker chemistry resulted in conjugates of the cross-linked “lattice-type” characterized by their chromatographic profiles, which are much larger than the “sun-type” uni- or bi-directional monomeric conjugate made by conjugating oligosaccharide to CRM_197_ [28,29,30,37]. In the mouse model, these were similar to or more immunogenic than those of a MenACWY-CRM_197_ conjugate vaccine, and it will be interesting to see if the very different molecular structures formed will affect the stability and/or immunogenicity of serogroups C, W and Y.

To achieve the goals established by the WHO Defeating Meningitis by 2030 initiative, the provision of affordable, multi-dose meningococcal vaccines that have sufficient coverage to eliminate those strains of bacteria that can be controlled through conjugate vaccines is required [38]. These vaccines must be able to be manufactured consistently in sufficient quantities to deliver to all the regions and countries where needed. The impact of MenAfriVac has demonstrated that meningococcal disease outbreaks can be potentially eliminated in sub-Saharan Africa with a pentavalent NmCV covering serogroups A, C, Y, W and X, and the consistency of production, and duration of protection following a single dose of MenAfriVac has allowed the vaccine to be rolled out to increasingly more countries, further eliminating disease spread and the possibility of escape mutations. NmCV-5 has been demonstrated to be high quality, stable, safe and immunogenic, and has the potential to build upon the progress made by MenAfriVac in eliminating meningococcal disease outbreaks in Africa. In addition, this may be a more affordable meningitis prevention tool for other areas of the world.

## 5. Materials and Methods

### 5.1. Materials

Purified bulk polysaccharides were in the form of lyophilized (for ^1^H-NMR, stored at 2–8 °C) and liquid samples (stored at −20 °C). Individual bulk conjugates were stored frozen at −20 °C. Freeze-dried vaccine in 1-dose and 5-dose presentations were stored at 2–8 °C. The vaccine was formulated with Tris buffer, sodium citrate and sucrose prior to freeze-drying. Either a saline diluent, or an aluminum phosphate (250 µg/mL aluminum) in saline diluent was used for reconstitution of the final vial. The final lots had a pH of 6.0–7.0 in saline or in adjuvanted saline. For a description and details of their manufacture see references [20,21].

### 5.2. O-acetylation 

^1^H-NMR spectroscopy was used to measure the structural identity and *O*-acetyl content of polysaccharides [39]. The spectrum of fully *O*-acetylated polysaccharide was compared to reference spectra to confirm the identity and determine if contaminants were present. Before ^1^H-NMR spectroscopy was performed, the samples were twice dissolved in D_2_O and lyophilized. The NMR spectroscopy was performed using a 700 MHz Bruker NEO AVANCE spectrometer (Bruker, Coventry, UK), fitted with a QCI-F cryoprobe. ^1^H-NMR spectra were collected using the *zg* pulse-sequence at 303.2 K, with a D1 of 26 s. The% *O*-acetyl contents were measured following base hydrolysis by integration of the *N*-acetyl and free acetate resonance signals. The uncertainty of the assay is ±4%.

The Hestrin method, a European Pharmacopoeia (E.P.) method, using *N*-acetylcholine as a standard, was also used. At SIIPL values were calculated in mmoles/g of polysaccharide which were further converted into% O-acetylation for comparison, using the equation, ((mmol *O*-acetyl/g polysaccharide × g/mol polysaccharide)/(0.95 × 1000 mmol/mol *O*-acetyl) × 100%) = mol *O*-acetyl/mol polysaccharide repeating unit × 100 = 100 = % *O*-acetylation, with sodium-coordinated monomer residue weights for typical lots of 340.89 (A), 343.51 (C), 494.30 (Y) and 490.09 (W), and an allowance of 5% by weight of impurities (nucleic acid, protein, salt, water or other impurities).

### 5.3. Molecular Size

For molecular size distribution (MSD) of vaccine materials, an HPLC-Size Exclusion Chromatography (SEC) method was used for polysaccharides and bulk conjugates. Fifty or 100 µg of polysaccharide, or protein in the case of bulk conjugates were loaded onto a TSKgel 6000_PWXL_ + 5000_PWXL_ column series with PWXL guard column, and eluted with PBS ‘A’ (10.1 mM Na_2_HPO_4_, 1.84 mM KH_2_PO_4_, 171 mM NaCl, 3 mM KCl, pH 7.3–7.5) at a flow rate of 0.25 mL/min for 120 to 150 min. Absorbance at 280 nm and 214 nm, and refractive index signals were monitored. Column marker elution time was monitored as a system suitability test, along with 0.25% ethylene glycol with respect to plate count and symmetry. Distribution coefficients (K_D_) were determined using elution times of the Vo (salmon DNA) and Vt (tyrosine) markers and the peak elution time and% eluting by specified K_D_ values, typically 0.50, were reported. The variability (precision) of the MSD method determined during method validation for bulk conjugates at NIBSC was ≤7.5% CV, and at SIIPL was ≤10% CV.

At SIIPL, size-exclusion chromatography with multi-angle light scattering (SEC-MALS) was also used to more accurately measure the molar mass of the bulk conjugates. Molar mass analysis was carried out using a SEC-MALS instrument with HPLC from Agilent Technologies (Santa Clara, CA, USA) with 18-angle laser light scattering detector (Heleos II) and refractive index detector (Optilab rEX), both from Wyatt Technology Corp. (Santa Barbara, CA, USA). A volume of 25 µL of bulk conjugate (>0.4 mg/mL)) loaded on to the Shodex column SB 803 + 806 HQ used in series and eluted with phosphate buffer (100 mM monobasic sodium phosphate, 7.7 mM sodium azide, pH 7.1–7.3). The Zimm formalism was used for Mw determinations M = R(0)/K c (dn/dc)^2^ where M is the molecular weight of the analyte, R(0) the reduced Rayleigh ratio (i.e., the amount of light scattered by the analyte relative to the laser intensity) determined by the MALS detector and extrapolated to angle zero, c the weight concentration determined by the UV or dRI detector, dn/dc the refractive index increment of the analyte (essentially the difference between the refractive index increment of the analyte and the buffer), and K, the system constant. In-house generated dn/dc values for each polysaccharide, 0.185 mL/g for tetanus toxoid carrier protein, and the literature value 0.1659 mL/g for CRM_197_ were used in calculating the weight-average molar mass concentration (Mw, g/mol) using ASTRA 7 software.

### 5.4. Endotoxin Content

To measure the endotoxin content of polysaccharides and final vials using LAL assays. The gel-clot method was used at NIBSC, according to the E.P. chapter 2.6.14 method B. The assay was standardised using the WHO 3rd IS for endotoxin (NIBSC code 10/178). A pharmacopeial kinetic turbidimetric assay (KTA) was used by SIIPL with a freeze-dried endotoxin standard control.

### 5.5. Protein Content

Bicinchoninic acid and Lowry methods were used for determining the protein content of CRM_197_ and TT conjugates respectively, using bovine serum albumin standards (cat 23209, Thermo Fisher Scientific, Waltham, MA, USA).

### 5.6. Free Saccharide

An ICS5000 High Performance Anion Exchange Chromatography with Pulsed Amperometric Detection (HPAEC-PAD) system equipped with AminoTrap and CarboPac PA-1 columns (Dionex, Thermo Fisher Scientific, Sunnyvale, CA, USA) was used to quantify the saccharide content for all serogroups in the conjugate vaccine lots. The drug product lots were depolymerised using HCl for groups C and X, or Trifluoroacetic acid (TFA) for groups A, Y and W) [40]. TFA hydrolysates were dried and reconstituted in water prior to chromatography. As bulk conjugates and final fills contained sucrose as a formulation sugar, a Microsep^®^ 3K filter (Pall Corporation, Port Washington, NY, USA) was used to replace sucrose with water before hydrolysis [41]. Validation of this step for a MenC-containing vaccine showed no loss of saccharide, free or total.

For separation of free from conjugated saccharide, at NIBSC, Microcon-30 (30 kDa cut off) or Microcon-100 ultrafiltration membrane devices (2 mL) were used for rCRM_197_ or TT conjugates, respectively [42]. Total saccharide hydrolysates were also filtered as a control for recovery. At SIIPL, a validated deoxycholate-HCl precipitation method was used [21]. During validation of these methods, method accuracy was assessed through the recovery of an oligosaccharide spike (ultrafiltration) or polysaccharide spike (DOC-HCl protein precipitation) with each serogroup saccharide achieving >70% or 70–130% recovery, respectively. WHO International Standards or in-house polysaccharide standards were used as quantitative standards over a range of 0.17 to 27 µg/mL [40]. Four µg/mL of fucose (Sigma F2252) for MenY, MenW and Men X, glucosamine-1-phosphate (G-9753) for MenA, or glucuronic acid (G-8645) for MenC, were added to samples and standards as internal controls just prior to chromatography.

Isocratic elution condition of 100 mM NaOH with 80 mM NaOAc with flow rate of 1 mL/min was used for MenC, with a running time of 25 min. For MenA, Y and W, a multi-step gradient elution was used as follows: 0–18 min, 15 mM NaOH; 18–42 min, 100 mM NaOH and 100–224 mM NaOAc; 42–52 min, 400 mM NaOH, 52–60 min, 15 mM NaOH with flow rate of 1 mL/min. Mannosamine-6-phosphate (MenA), sialic acid (MenC), galactose (MenW), and glucose (MenY) were detected by pulsed amperometric detector using quadruple-potential waveform with 2 Hz data collection rate. For MenX, a different multi-step gradient elution was used: 0–13 min, 50 mM NaOH; 13–25 min, 50 mM NaOH and 100–224 mM NaOAc; 25–35 min, 400 mM NaOH, 35–40 min, 50 mM NaOH with flow rate of 1 mL/min. Chromeleon software (versions 6.8 and 7.2, Dionex, Thermo Fisher Scientific, Sunnyvale, CA, USA) was used to program the runs and analyse data. Further details are available in references [40,42]. The variability (precision) of the% free saccharide values determined at validation for bulk conjugates at NIBSC was less than ±2% free saccharide for serogroups A,Y,W and X, and ±3.3% free saccharide for serogroup C, and at SIIPL was ≤15% CV.

To reduce non-specific interference during the quantitation of MenC and MenA due to co-elution of serogroups of similar structure, namely the sialic acid of W and Y (co-eluting with the sialic acid of MenC) and group × (with A), either of two approaches were used: using a mixture of polysaccharides standards, or applying experimentally-determined corrections (6.9% of sialic acid arising from MenW and 2.8% of sialic acid arising from MenY were subtracted from the MenC content; and an 8% contribution of HexN-P arising from MenX was subtracted from MenA).

### 5.7. Adjuvant Adsorption

The degree of adsorption of the conjugates to the aluminum phosphate was measured for the Phase 1 drug product lot. Duplicate samples that had been reconstituted in saline or adjuvant were held for 4 h at 25 °C and the supernatants following centrifugation at 8500× *g*, 15 min and the saccharide content in the supernatants was measured.

### 5.8. Mouse Immunogenicity

Groups of eight BALB/c mice received three 0.2 mL subcutaneous injections containing 1/10 single human dose (SHD) (0.5 µg each polysaccharide) of an R&D NmCV-5 lot with or without 1/10 SHD aluminum phosphate adjuvant at 0, 14 and 28 days. A control group received 1/10 SHD of a licensed ACWY-CRM_197_ conjugate vaccine. Sera were collected at 14 days post-each immunization by tail or terminal bleed and assessed for anti-meningococcal polysaccharide IgG content by ELISA. For the ELISA, plates were coated with 5 µg/mL of individual meningococcal polysaccharides (A, C, Y, W or X) manufactured at SIIPL and mixed with 5 ug/mL methylated human serum albumin. Plates were blocked with PBS containing fetal calf serum and Brij-35. Reference sera were comprised of pooled sera produced in mice following administration of 3 doses of NmCV-5 and assigned arbitrary concentrations (units/mL) for anti-A, C, Y, W and X IgG. Immune sera and reference sera were added at 1/100 dilution, and 2-fold dilutions were made. Negative control sera were from mice or rabbits immunised with saline only. The amount of IgG bound was detected using an anti-mouse monoclonal antibody-HRP or a goat-anti-rabbit IgG-HRP. Analysis was performed using European Directorate for the Quality of Medicine CombiStats Software Version 5.0 and used a four-parameter logistic model (sigmoid curves) in order to determine the potencies of study samples relative to the reference sera [43]. Statistical significance was measured using Minitab software (V17) for one-way ANOVA comparisons of natural-log transformed IgG concentrations, and Tukey’s 95% simultaneous confidence intervals.

### 5.9. Rabbit Immunogenicity

An immunogenicity study was performed to evaluate the potential of NmCV-5 produced for the Phase 1 (with and without adjuvant) and Phase 3 clinical trials (without adjuvant). The study was done in New Zealand White rabbits, eight animals per group following three intramuscular immunizations (5 µg for each serotype) at 14 day intervals. Immune responses were assessed by measuring the IgG and bactericidal antibodies to Men A, C, Y, W, and X in rabbit serum samples collected on Days 0 (pre-immune), 28 (post-2) and 35 (post-3 sera).

Antibody titers were determined using a multiplex bead-based IgG immunoassay and serum bactericidal assay (rSBA) using rabbit complement for meningococcal serogroups A, C, Y, W, and X.

For the multiplexed bead-based IgG immunoassay, meningococcal polysaccharides A, C, Y, W and X were supplied by Serum Institute of India Pvt Ltd. (Pune, India) and used in bead coupling. The individual meningococcal polysaccharide (A, C, Y, W, X)-bead reagents for the assay were prepared by covalently coupling each polysaccharide to carboxylated beads (Luminex™, Austin, TX, USA). Serial two-fold dilutions of an internal reference serum and the pre- and post-immunization sera samples were incubated with PS-coupled beads. R-phycoerythrin-conjugated goat anti-rabbit IgG polyclonal antibody (Jackson ImmunoResearch, West Grove, PA, USA) was added to detect serogroup specific antibody bound to PS coupled beads. The median fluorescence intensity (MFI) units for gated singlet bead events were measured using a semi-automated flow cytometry instrument (Bio-Plex200, Bio-Rad Laboratories, Hercules, CA, USA) with Bio-Plex Manager software. The antibody titers were determined as the reciprocal of the highest dilution of the serum sample that had ≥100 MFI (median fluorescence intensity) after subtraction of background MFI. Titers that fell below the lower limit of detection were assigned a value of ‘50’ for calculation of geometric mean titers (GMTs).

To determine rSBA titers, individual rabbit sera were inactivated at 56 °C before initiating the assay. The complement source was 3–4 week old rabbit complement (Pel-freez Biologicals, Rogers, AR, USA). The bacterial strains used in the rSBA assays were: serogroup A, CDC F8238, phenotype A:4:P1.9, O-Acetylation OAc+; serogroup C, strain C11, C:16:P1.7–1,1.1, OAc+; serogroup Y, strain M03 241125/S1975, Y:2a:P1.5,1.2, OAc−; serogroup W, M01 240070, W:NT:P1.18–1,1.3 and serogroup X, BF2/97. SBA titers were expressed as the reciprocal serum dilution yielding ≥50% killing after 60 min incubation at 37 °C. More than four-fold difference in titers was considered to be significant.

Statistical significance was measured using one-way ANOVA comparisons based on IgG and SBA titers, and Tukey’s 95% simultaneous confidence intervals.

## Patents

R.M.D., S.S.P., A.D.S. & N.A. are listed as inventors on patents/patent applications (PCT/IB2013/050739, IN201922019720, US9283270) covering pentavalent (A, C, W, X, Y) meningococcal conjugate vaccine (NmCV-5) composition, all assigned to SIIPL. A.M., S.K., P.S., S.K.G., S.G. are listed as inventors on several patents/patent applications covering pentavalent (A, C, W, X, Y) meningococcal conjugate vaccine (NmCV-5) related assays, all assigned to SIIPL.
